# 704 Extubation Success in Pediatric Burn Patients with Neuromuscular Blockade: A 10-year review

**DOI:** 10.1093/jbcr/irad045.180

**Published:** 2023-05-15

**Authors:** Noah Pierzchajlo, Richard Cartie

**Affiliations:** Mercer University of Medicine, Savannah, Georgia; Burn and Reconstructive Centers of America, Augusta, Georgia; Mercer University of Medicine, Savannah, Georgia; Burn and Reconstructive Centers of America, Augusta, Georgia

## Abstract

**Introduction:**

The use of neuromuscular blockade in ventilated pediatric burn patients is controversial and varied. While standard of care, including neuromuscular blockade in pediatric patients, has been long established for the non-burned population, no such literature exists for burns. We analyzed use of neuromuscular blockade in pediatric burn patients and its association of mechanical intubation and extubation success.

**Methods:**

We reviewed all pediatric patients admitted to the burn and wound care ICU who were intubated for more than 24 hours. Patient data was collected from 2012 through 2022. 442 patients were initially identified and 142 remained after removing screen failure. We compared patients based on administration of neuromuscular blockade (n=125).

**Results:**

Mortality (n=7) and reintubation (n=7) rates were not significantly affected by use of neuromuscular blockade. Patients who received blockade were also not significantly more likely to develop acute respiratory distress syndrome (ARDS) or pulmonary hypertension (HTN). Length of stay (LOS) in the hospital, intensive care unit (ICU), and time on the ventilator were all significantly increased with patients who received neuromuscular blockade. Patients who received blockade also had a significantly higher total burn surface area (TBSA). Conversely, patients who did not receive blockade were significantly more likely to be older.

**Conclusions:**

Our data shows that mortality and the need for reintubation is not significantly increased with the use of neuromuscular blockade, nor are the complications of prolonged paralysis such as ARDS and pulmonary HTN. LOS in the hospital, ICU, and time on the ventilator were expectedly increased with blockade, although these patients carried more injury than their non-paralyzed counterparts.

**Applicability of Research to Practice:**

This project illustrates how the use of neuromuscular blockade can be implemented safely and effectively in the critical care setting.

